# Incidence of Pediatric Cannabis Exposure Among Children and Teenagers Aged 0 to 19 Years Before and After Medical Marijuana Legalization in Massachusetts

**DOI:** 10.1001/jamanetworkopen.2019.9456

**Published:** 2019-08-16

**Authors:** Jennifer M. Whitehill, Calla Harrington, Cheryl J. Lang, Michael Chary, Waqaas A. Bhutta, Michele M. Burns

**Affiliations:** 1 Department of Health Promotion and Policy, University of Massachusetts Amherst, Amherst; 2 Massachusetts Department of Public Health, Division of Violence and Injury Prevention, Boston; 3 Regional Center for Poison Control and Prevention, Boston, Massachusetts; 4 Harvard Medical School, Boston, Massachusetts; 5 Division of Emergency Medicine, Boston Children’s Hospital, Boston, Massachusetts

## Abstract

**Importance:**

Pediatric health care contacts due to cannabis exposure increased in Colorado and Washington State after cannabis (marijuana) policies became more liberal, but evidence from other US states is limited.

**Objective:**

To document the incidence of pediatric cannabis exposure cases reported to the Regional Center for Poison Control and Prevention (RPC) before and after medical marijuana legalization (MML) in Massachusetts.

**Design, Setting, and Participants:**

Cross-sectional comparison of pediatric cannabis exposure cases 4 years before and after MML in Massachusetts. The exposure cases included those of 218 children and teenagers aged between 0 and 19 years, as reported to the RPC from 2009 to 2016. Census data were used to determine the incidence. Data analysis was performed from November 12, 2018, to July 20, 2019.

**Exposure:**

Cannabis products.

**Main Outcomes and Measures:**

Incidence of RPC-reported cannabis exposure cases, both single substance and polysubstance, for the age group of 0 to 19 years, and cannabis product type, coingestants, and clinical effects.

**Results:**

During the 8-year study period (2009-2016), the RPC received 218 calls involving cannabis exposure (98 single substance, 120 polysubstance) in children and teenagers aged 0 to 19 years, representing 0.15% of all RPC calls in that age group for that period. Of the total exposure cases, males accounted for 132 (60.6%) and females 86 (39.4%). The incidence of single-substance cannabis calls increased from 0.4 per 100 000 population before MML to 1.1 per 100 000 population after (incidence rate ratio, 2.4; 95% CI, 1.5-3.9), a 140% increase. The age group of 15 to 19 years had the highest frequency of RPC-reported cannabis exposures (178 calls [81.7%]). The proportion of all RPC calls due to single-substance cannabis exposure increased overall for all age groups from 29 before MML to 69 afterward. Exposure to edible products increased after MML for most age groups.

**Conclusions and Relevance:**

Pediatric cannabis exposure cases increased in Massachusetts after medical marijuana was legalized in 2012, despite using childproof packaging and warning labels. This study provides additional evidence suggesting that MML may be associated with an increase in cannabis exposure cases among very young children, and extends prior work showing that teenagers are also experiencing increased cannabis-related health system contacts via the RPC. Additional efforts are needed to keep higher-potency edible products and concentrated extracts from children and teenagers, especially considering the MML and retail cannabis sales in an increasing number of US states.

## Key Points

**Question:**

Does the number of pediatric cannabis (marijuana) exposure cases before medical marijuana legalization in Massachusetts differ from the number afterward?

**Findings:**

In this repeated cross-sectional study, there were 218 calls related to cannabis exposure received by the Regional Center for Poison Control and Prevention in Massachusetts, constituting 0.15% of all calls concerning children and teenagers aged 0 to 19 years. The number of calls regarding single-substance cannabis exposure, especially edible product exposure, in this age group showed a statistically significant increase; the number of such calls for all cannabis product types more than doubled from 29 calls 4 years before MML to 69 calls 4 year afterwards.

**Meaning:**

This study suggests that states liberalizing marijuana policies should consider strengthening regulations to prevent unintentional exposure among young children and enhancing efforts to prevent use by teenagers, with particular attention to edible cannabis products and concentrated extracts.

## Teaser

This cross-sectional study investigates the number of pediatric cannabis exposure cases reported to a regional poison control center before vs after medical marijuana legalization in Massachusetts.

## Introduction

Cannabis (marijuana) has been legalized for medical use in 33 US states and for adult use (ie, recreational use) in 11 as of July 2019.^1^ Evidence from the first US states to liberalize and commercialize cannabis suggests that such legalization policies may lead to unintended consequences for youth including increase in poisoning from unintentional or intentional exposure.^2,3^

For young children aged 0 to 9 years, unintentional exposure to cannabis owned or used by other members of the household is of concern. Data from Colorado and Washington State indicate that, for children aged 0 to 9 years, the number of cannabis-related calls to the regional poison center increased as commercial medical marijuana dispensaries proliferated^4^ and after cannabis use was legalized for general adult (recreational) purposes.^5,6,7^ Edible cannabis products were involved in nearly half of the cases with a known product type.^5,7,8^

For older children and teenagers aged 10 to 19 years, acute illness or severe psychological effects related to intentional cannabis use may result in a health care contact. Adolescent urgent care and emergency department visits related to cannabis increased in Colorado between 2005 and 2015 as cannabis laws became more permissive^9^ despite the fact that population surveys generally have not found increases in the prevalence of adolescent cannabis use.^10,11^ Most prior research on medical marijuana policies and pediatric exposures has been conducted in the western United States, which may have different cultural and policy environments compared to other regions.

Massachusetts voters passed medical marijuana legalization (MML) by ballot initiative in 2012 and the first dispensaries opened in 2015. Medical marijuana was initially regulated by the Massachusetts Department of Public Health, which imposed strict requirements for childproof packaging, warning labels, and dosage information, and prohibited the manufactured edible products from resembling commercially available candy.^12^ These regulations were generally more restrictive than those passed in the first states to commercialize medical marijuana. In December 2018, oversight was transferred to the Cannabis Control Commission, a new state agency. Although medical marijuana is primarily used by adults, it is available for patients younger than 18 years if diagnosed with a debilitating, life-limiting illness or condition for which benefits outweigh potential risks as determined by 2 Massachusetts-licensed, certifying physicians.^13^ Patients receiving medical marijuana are able to receive up to a 60-day supply of marijuana at a time, defined as 283.5 g of dry flower or 42.5 g of cannabis concentrates or resin.^13^ This amount is a potentially greater quantity than allowed in other states, which generally have 28- to 56-g limits.^14,15^

The purpose of this study was to compare cannabis exposure calls for children and teenagers aged 0 to 19 years received by the Regional Center for Poison Control and Prevention (RPC) for 4 years before and after MML in Massachusetts. Specifically, we sought to (1) describe the incidence of single-substance and polysubstance cannabis-involved exposures for children and teenagers; (2) examine whether cannabis-related calls increased as a proportion of all RPC calls; and (3) assess patterns in product formulations involved in RPC-reported cases. Understanding and learning from trends in cannabis exposures and coexposures after MML was implemented can have implications for policy makers in states now implementing medical or nonmedical marijuana legalization.

## Methods

This study followed the Strengthening the Reporting of Observational Studies in Epidemiology (STROBE) reporting guideline. For this cross-sectional study, we obtained data on all Massachusetts cannabis exposure cases reported to the Massachusetts and Rhode Island RPC between January 1, 2009, and December 31, 2016. We focused on the pediatric age range of 0 to 19 years, excluding cases of unknown age. Data were obtained from the RPC’s local installation of the American Association of Poison Control Centers’ data warehouse, the National Poison Data System (NPDS). The RPC staff reviewed the cases, and only deidentified data were shared with external collaborators. The study received an exempt determination from the institutional review board at Boston Children’s Hospital, Boston, Massachusetts, owing to the use of deidentified patient data.

Data analysis was performed from November 12, 2018, to July 20, 2019. Data included age, sex, route of exposure (inhalation/nasal, ingestion, rectal, multiple routes, or unknown), caller location (health care facility, own residence, school, or other), exposure site (own residence, other residence, school, or unknown), medical outcome, and intent. Medical outcomes were classified using the published NPDS definitions (death; major effect; moderate effect; no effect; unable to follow, judged as potentially toxic exposure; not followed, judged as nontoxic exposure; not followed, minimal clinical effects possible; or unrelated effect).^16^ For reporting, we combined the latter 3 categories into 1 category for cases not followed, with minimal or unrelated effects. Intent was classified into 8 categories that were grouped as intentional (abuse and misuse, suspected suicide, or unknown intentional), unintentional, other (malicious or contamination/sampling), adverse drug reaction, or unknown. The RPC also provided aggregate numbers of all poisoning exposure calls in Massachusetts by age group and year.

Cannabis-related cases were identified using the product code based on the NPDS. We classified these product codes into the following 4 categories: plant, edible preparations, concentrated extracts, and other. The plant category included codes for generic marijuana dried plants (code 083000) as well as undried plants (310123). The code for edible preparation (310121) constituted its own category. To include oils, tinctures, and vaping products, which we classified under concentrated extracts, we included specific marijuana product codes for the following subcategories: concentrated extracts, including oils and tinctures (code 310124); marijuana device with added flavor (310034), without added flavor (310033), and flavor unknown (310096); as well as electronic cigarettes (e-cigarettes): marijuana liquid with added flavors (310036), without added flavors (310035), and flavor unknown (310097). Other marijuana product codes included the following: marijuana oral capsule or pill preparation (code 310122), marijuana other or unknown preparation (310126), marijuana pharmaceutical preparation (200618), and marijuana topical preparation (310125).

Marijuana codes have changed in the NPDS over time in ways relevant to this analysis. The generic marijuana code for dried plant material (code 083000) was the first marijuana code in the NPDS and was activated in 1985. A code for pharmaceutical preparation (200618) was activated in 2010. Codes related to marijuana vaping (e-cigarette) products (310096, 310034, 310033, 310097, 310036, and 310035) were added in 2014. In 2016, codes were added for marijuana concentrated extract (310124), edible preparation (310121), oral capsules or pill preparation (310122), topical preparation (310125), undried plant (310123), and other or unknown preparation (310126). Two of us (C.J.L. and W.A.B.) recoded data for 2009 to 2016 into the newer codes to allow analysis by product type for the time before these codes were used. The procedures were discussed with a specialist in poison information—one of the trained clinicians who answer calls to the RPC—to ensure that the coding would be consistent with current coding practices when fielding calls. For example, the authors reviewed case notes from cases previously coded as generic marijuana (083000); if the case notes referred to a cannabis exposure from an edible form of marijuana, they recoded the data using the new code for edible preparation (310121). Ambiguous cases were rare and were discussed with the RPC medical director (M.M.B.). Plant-related cases generally involved dried plant material with 2 cases of exposure to undried plants.

To determine whether cannabis exposure substantially contributed to the major clinical outcomes for each case, a group of 3 medical toxicologists independently reviewed each case. Inter-rater agreement was quantified by the Cohen κ, with a value of 0.49. Guidelines for defining major clinical outcomes were formulated based on a 10% random sample, which was not used for subsequent calculations of agreement. We considered a reported cannabis ingestion to be clinically significant if the symptoms that warranted medical intervention or evaluation were more consistent with the known effects of cannabis than any other substances reported to be ingested. For example, we did not consider the ingestion of cannabis to be clinically significant in a patient presenting with somnolence and conjunctival injection and reported cannabis and alcohol ingestion. Although conjunctival injection is consistent with cannabis ingestion, the somnolence is the clinically significant factor and is more likely due to alcohol ingestion. In contrast, we considered the ingestion of cannabis to be clinically significant in a patient presenting with paranoia and reported ingestion of marijuana and alcohol.

### Statistical Analysis

Cases were separated into 2 categories: single-substance cannabis exposures (ie, cannabis was the only substance involved) and polysubstance cases (ie, cannabis was one of several substances). The medical marijuana law took effect in Massachusetts on January 1, 2013, so the proportion of all calls to the RPC involving cannabis only or cannabis plus other substances was assessed for statistical significance before (2009-2012) vs after (2013-2016) MML.

We stratified the analyses by age group (0-4, 5-9, 10-14, and 15-19 years). The incidence was calculated by dividing the number of single-substance and polysubstance cases by the number of person-years contributed by all individuals in the Massachusetts population during the time period (4 years pre-MML and 4 years post-MML) and multiplying by 100 000. Population estimates by age were obtained from the US Census data for 2009 to 2016.^17,18^ We computed and compared the incidence rate ratios (IRRs) before vs after MML with 95% CIs. We also compared the proportion of all RPC calls that were related to cannabis before vs after MML. We stratified single-substance cannabis cases by formulation and compared each age group’s number and proportion of RPC-reported cases of each product type before vs after MML. For comparisons of proportions, Fisher exact tests were used when cells in the 2 × 2 table had 5 or fewer cases; otherwise, χ^2^ tests were used. All statistical analyses were performed using Stata software, version 15 (StataCorp LP). The significance threshold was α = .05, and all testing was 2-sided.

## Results

During the 8-year study (2009-2016), a total of 218 calls received by the RPC were related to cannabis exposure among children and teenagers aged 0 to 19 years, representing 0.15% of all RPC calls in that age group for that period. The age group of 15 to 19 years had the highest frequency of RPC-reported cannabis exposures (178 calls [81.7%]). Of the 218 exposure cases, males accounted for 132 (60.6%) and females 86 (39.4%). Males accounted for 51 of the 98 single-substance cannabis cases (52.0%) and 81 of the 120 polysubstance cases (67.5%) (Table 1). Teenagers aged 15 to 19 years were involved in the highest number of cases, accounting for 69 cannabis-only exposures (70.4%) and 109 polysubstance exposures (90.8%). The youngest children, aged 0 to 4 years, accounted for 19 single-substance cannabis cases (19.4%) and 3 polysubstance cases (2.5%).

**Table 1.** Cannabis-Related Cases Reported to the Massachusetts Regional Center for Poison Control and Prevention for Patients Aged 0 to 19 Years, 2009-2016

Characteristic	Cases, No. (%)		
	Cannabis Single-Substance (n = 98)	Cannabis-Involved Polysubstance (n = 120)	All Cannabis-Involved (N = 218)
Sex			
Female	47 (48.0)	39 (32.5)	86 (39.4)
Male	51 (52.0)	81 (67.5)	132 (60.6)
Age, y			
0-4	19 (19.4)	3 (2.5)	22 (10.1)
5-9	2 (2.0)	2 (1.7)	4 (1.8)
10-14	8 (8.2)	6 (5.0)	14 (6.4)
15-19	69 (70.4)	109 (90.8)	178 (81.7)
Route of exposure			
Inhalation/nasal	50 (51.0)	103 (85.8)	153 (70.2)
Ingestion	46 (46.9)	8 (6.7)	54 (24.8)
Inhalation/nasal/ingestion	0	2 (1.7)	2 (0.9)
Rectal	1 (1.0)	0	1 (0.5)
Unknown	1 (1.0)	7 (5.8)	8 (3.7)
Intention of exposure			
Intentional, abuse and misuse	62 (63.3)	77 (64.2)	139 (63.8)
Intentional, suspected suicide	0	30 (25.0)	30 (13.8)
Intentional, unknown	3 (3.1)	4 (3.3)	7 (3.2)
Unintentional^a^	25 (25.5)	6 (5.0)	31 (14.2)
Other, malicious	4 (4.1)	0	4 (1.8)
Other or contamination, sampling	3 (3.1)	0	3 (1.4)
Adverse reaction, drug	0	1 (0.8)	1 (0.5)
Unknown	1 (1.0)	2 (1.7)	3 (1.4)
Medical outcome			
Death	0	0	0
Major	1 (1.0)	3 (2.5)	4 (1.8)
Moderate	34 (34.7)	66 (55.0)	100 (45.9)
Minor	20 (20.4)	34 (28.3)	54 (24.8)
None	7 (7.1)	4 (3.3)	11 (5.0)
Unable to follow, judged potentially toxic	30 (30.6)	9 (7.5)	39 (17.9)
Not followed, minimal or unrelated	6 (6.1)	4 (3.3)	10 (4.6)

^a^ Unintentional exposure category includes general, environmental, misuse, and unknown.

Inhalation or nasal exposure was the most common route and was observed in 50 single-substance cannabis cases (51.0%) and 103 polysubstance cases (85.8%). An additional 46 cannabis-only cases (46.9%) resulted from ingestion. Approximately two-thirds of the single-substance cannabis cases (65 [66.3%]) and 111 polysubstance cases (92.5%) were classified as intentional exposures. Among the cases involving children aged 0 to 4 years, most (19 [86.4%]) were due to ingestion (eTable in the Supplement), whereas for teenagers aged 15 to 19 years, 140 cases (78.7%) were related to inhalation. Most calls (172 [78.9%]) were received from health care facilities and 37 (17.0%) from the caller’s own residence (eTable in the Supplement).

Moderate and minor effects were the most common medical outcomes reported by RPC professionals, constituting 154 cases (70.6%) (Table 1). No deaths were reported. A higher proportion of cases (3 [2.5%]) was observed involving a major effect for polysubstance cases (as many as 8 substances involved) compared with cases involving only cannabis. The single RPC call for a major effect from single-substance cannabis exposure involved 2 unrelated adolescent males (aged 17 and 18 years) with no known health problems who collapsed while playing sports, hours after smoking what they believed to be marijuana. There was no report of intentional ingestion of “synthetic marijuana.” They were found to be in cardiac arrest and successfully resuscitated. A sample of the substance they used was not available for analysis. This case suggests the possibility of contaminated marijuana as the cause of the clinical effects.

For polysubstance cases, the following were the most frequently coingested substances were alcohol (26%); 3,4-methylenedioxy-methamphetamine (MDMA/ecstasy) (11%); preparations containing dextromethorphan (9%); benzodiazepines (8%); and crack/cocaine (5%). Of the 120 cases, medical toxicologists classified 85 (71%) as cases where cannabis ingestion likely had clinical importance, 21 (18%) as cases where cannabis ingestion likely had no clinical significance, and 14 cases (12%) as unclear. In cases in which medical toxicologists judged that cannabis had a substantial impact on the clinical presentation, the other substances most frequently ingested were alcohol (n = 22 [26%]), MDMA (n = 8 [9%]), alprazolam, a benzodiazepine sold as Xanax (n = 7 [8%]), and preparations containing dextromethorphan (n = 6 [7%]).

The incidence for all cases involving cannabis for the 4 years before and after MML was 1.3 per 100 000 population and 2.2 per 100 000 population, respectively (IRR, 1.7; 95% CI, 1.2-2.2). The incidence of cannabis single-substance cases was 0.4 per 100 000 population before MML and 1.1 per 100 000 population after (IRR, 2.2; 95% CI, 1.5-3.9), a 114% increase (Table 2). The incidence of cannabis-involved polysubstance cases was 0.8 per 100 000 population before MML and 1.0 per 100 000 population after (IRR, 1.2; 95% CI, 0.9-1.8).

**Table 2.** Pediatric Cannabis Exposure Calls to the Massachusetts Regional Center for Poison Control and Prevention Before vs After MML

Age Group, y	No. of Calls (Incidence per 100 000 Population)					
	Cannabis Single-Substance Cases			Cannabis-Involved Polysubstance Cases		
	Before MML, 2009-2012	After MML, 2013-2016	After vs Before MML, IRR (95% CI)	Before MML, 2009-2012	After MML, 2013-2016	After vs Before MML, IRR (95% CI)
All, 0-19	29 (0.4)	69 (1.1)	2.4 (1.5-3.9)	54 (0.8)	66 (1.0)	1.2 (0.9-1.8)
0-4	6 (0.4)	13 (0.9)	2.2 (0.8-7.0)	0 (0.0)	3 (0.2)	NC^a^
5-9	0 (0.0)	2 (0.1)	NC^a^	0 (0.0)	2 (0.1)	NC^a^
10-14	1 (0.1)	7 (0.4)	7.1 (0.9-321.3)	4 (0.2)	2 (0.1)	0.5 (0.1-3.6)
15-19	22 (1.2)	47 (2.6)	2.2 (1.3-3.8)	50 (2.7)	59 (3.2)	1.2 (0.8-1.8)

Abbreviations: IRR, incidence rate ratio; MML, medical marijuana legalization; NC, not calculated.

^a^ The IRR cannot be calculated when incidence is 0 cases.

When stratified by age, the incidence of both single-substance and polysubstance cannabis cases was highest for teenagers aged 15 to 19 years (Table 2). The incidence rate more than doubled from before MML to after for this age group (IRR, 2.11; 95% CI, 1.24-3.66). This finding was consistent with increases in incidence for the other age groups, although the IRRs for other age groups did not reach statistical significance, likely owing to limited power from the small numbers of cases.

Over time and across age groups, most cannabis-related calls involved dried plant material. Among children aged 0 to 4 years, compared with the time before MLL, a statistically significant increase was observed in the incidence of exposures to edible products after MML (Figure). Among teenagers aged 15 to 19 years, a statistically significant increase was observed in the incidence of exposures to concentrated products and an increase in edible product exposures that was at the significance threshold was found (*P* = .05).

**Figure.**
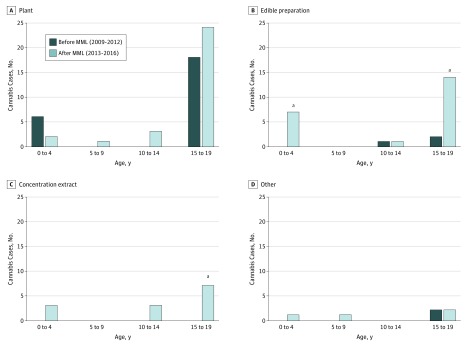
Regional Center for Poison Control and Prevention–Reported Pediatric Single-Substance Cannabis Exposure Cases by Product Type 4 Years Before vs After Medical Marijuana Legalization (MML) in Massachusetts ^a^Indicates a statistically significant change in the percentage of all Regional Center for Poison Control and Prevention calls from before to after MML.

The proportion of RPC cases due to cannabis (as a single substance) increased for all of the youngest children (0-4 years) from 6 to 13 cases, and for teenagers (10-19 years) from 23 to 54 cases after MML compared with those in the prior period (Table 3). Although cannabis exposures represented a small proportion of the cases handled by the RPC—0.15% of the overall cases for children and teenagers aged 0 to 19 years—the proportion doubled for each age group after MML.

**Table 3.** Proportion of Pediatric Regional Center for Poison Control and Prevention Cases Involving Cannabis Before vs After MML in Massachusetts

Age Group, y	No. of Cannabis Cases (% of All Massachusetts RPC Cases)					
	Cannabis Single-Substance Cases			Cannabis-Involved Polysubstance Cases		
	Before MML, 2009-2012	After MML, 2013-2016	*P* Value for Difference^a^	Before MML, 2009-2012	After MML, 2013-2016	*P* Value for Difference^a^
All, 0-19	29 (0.04)	69 (0.10)	<.001	54 (0.07)	66 (0.10)	.07
0-4	6 (0.01)	13 (0.03)	.05	0	3 (0.01)	.10
5-9	0	2 (0.02)	.22	0	2 (0.02)	.22
10-14	1 (0.01)	7 (0.11)	.03	4 (0.06)	2 (0.03)	.47
15-19	22 (0.25)	47 (0.57)	<.01	50 (0.58)	59 (0.72)	.27

Abbreviations: MML, medical marijuana legalization; RPC, Regional Center for Poison Control and Prevention.

^a^ *P* values from χ^2^ tests or Fisher exact test when cell sizes were less than 5.

## Discussion

This analysis of RPC-reported cannabis exposures reveals a significant increase in the overall incidence of pediatric exposures after MML in Massachusetts. The increase occurred despite the cannabis product packaging being designed to be difficult for young children to open, being unappealing to the youth, and requiring warning labels instructing that the product be kept away from children.

Our study also highlights the fact that, in addition to concerns about unintentional cannabis ingestion among young children, adolescents aged 15 to 19 years are experiencing negative cannabis-related consequences that result in health care contacts via the poison control system. These findings are consistent with findings from other states and the continued observation of the unintended consequences of MML and its influence on the pediatric population.^7^ Follow-up studies should assess whether Massachusetts, like Colorado and Washington, may see a continuing increase in pediatric cannabis exposure cases as implementation of commercial, adult-use cannabis sales continues. States that have not revised their cannabis laws do not appear to be experiencing increases in cannabis exposures.^19^

Medical outcomes of single-substance exposures were generally moderate to minor, but the increasing prevalence of exposures to concentrated and edible cannabis products and decrease in exposures to plant material is concerning because of the increased potency of these product types. Prior studies have been limited in examining product formulations prior to 2010 because a single generic product code for marijuana was used until that time. In addition, the number of codes was expanded in 2014 to include vaping products and in 2016 to include edibles as products available in legal markets. We overcame this limitation by having trained RPC personnel recode the data to use the new codes. Our finding that RPC-reported exposures to edible and concentrated cannabis products increased after MML is consistent with data from other states.^5^ This outcome is concerning considering recent evidence regarding associations between edible and concentrated cannabis and acute psychiatric and emergency department visits.^20^

There are several specific regulations that Massachusetts has recently adopted in the context of legalization for adult use that may help reduce cannabis poisoning in youth, including (1) laws requiring locked storage of more than 28 g (1 oz) of marijuana; (2) regulations requiring that cannabis packaging be in compliance with the US Consumer Product Safety Commission’s poison prevention packaging requirements^13^; and (3) fines or imprisonment for providing cannabis products or accessories to underaged individuals.^21^ In-home cannabis storage practices and fines for diversion to minors should be tracked to evaluate the extent to which these protections are implemented. Further, multistate studies that examine variation in marijuana regulations like those outlined above are needed to help determine the influence of specific policies and regulations and develop evidence-based recommendations.

### Limitations

This study has limitations. First, it cannot be discerned whether our finding of increased cannabis exposure calls to the RPC after MML is the result of increased exposures or reduced stigma for parents, caregivers, or adolescents calling to report such an exposure or going to the hospital.^22^ However, cannabis possession has been decriminalized in Massachusetts since 2010; consequently, during most of the prelegalization period, admitting marijuana use or possession would not have incurred legal ramifications. It is possible that policy changes or population-level trends not measured in this analysis contribute to the observed changes in the outcomes. Small numbers may have limited our power to detect statistically significant changes. The assessment of cannabis exposure is generally self-reported in RPC data and therefore potentially subject to recall bias or incomplete ascertainment of cases. Exposures may not be verified by laboratory testing. While this study is representative of Massachusetts and echoes findings from other states, trends in pediatric cannabis exposures may differ in other states.

Despite these limitations, this study adds to the evidence base regarding the potential influence of marijuana-related policies on youth. It will be important for Massachusetts and other states to continue to monitor poison center calls as cannabis policies are implemented. Such monitoring could be facilitated by creating state or national databases in which information about and package images for specific cannabis products involved in acute health care visits can be uploaded or by adding such information to an existing resource, such as the national Toxicology Investigators Consortium database.^23^ Creation of such databases could help regulators and public health officials understand any emerging patterns in problems from specific products or product types.

## Conclusions

In Massachusetts, the incidence of pediatric single-substance cannabis cases and the proportion of all pediatric cannabis-related calls to the RPC more than doubled after MML compared with before MML. States implementing medical marijuana policies should ensure that regional poison centers and other health care facilities are adequately prepared to respond to such increases. While it is encouraging that cannabis-related RPC calls were relatively uncommon for very young children, who may be unintentionally ingesting cannabis products owned by their parents, older siblings, or caregivers, continued efforts are needed to help keep cannabis away from them. It is also imperative to sustain efforts to help teenagers avoid cannabis use.

## Notes

Supplement. **eTable.** Characteristics of Cannabis-Related Calls to the Regional Poison Control Center by Age Group, Massachusetts, 2009-2016
