# Supplementary material for: Incidence of Pediatric Cannabis Exposure Among Children and Teenagers Aged 0 to 19 Years Before and After Medical Marijuana Legalization in Massachusetts
Source: JAMA Netw Open. 2019 Aug 16;2(8):e199456. doi: 10.1001/jamanetworkopen.2019.9456 (PMC6704738; doi:10.1001/jamanetworkopen.2019.9456)
Supplement: Supplement. — eTable. Characteristics of Cannabis-Related Calls to the Regional Poison Control Center by Age Group, Massachusetts, 2009-2016 [file jamanetwopen-2-e199456-s001.pdf]

Supplementary Online Content

Whitehill JM, Harrington C, Lang CJ, Chary M, Bhutta WA, Burns MM. Incidence of pediatric cannabis exposure among children and teenagers aged 0 to 19 years before and after medical marijuana legalization in Massachusetts. *JAMA Netw Open.* 2019;2(8):e199456. doi:10.1001/jamanetworkopen.2019.9456

eTable. Characteristics of Cannabis-Related Calls to the Regional Poison Control Center by Age Group, Massachusetts, 2009-2016

This supplementary material has been provided by the authors to give readers additional information about their work.

© 2019 Whitehill JM et al. *JAMA Network Open.*

eTable 1. Characteristics of Cannabis-Related Calls to the Regional Poison Control Center by Age Group, Massachusetts, 2009-2016

	Age group (years)									
	0 to 4 n=22		5 to 9 n=4		10 to 14 n=14		15 to 19 n=178		Total n=219	
	n	(%)	n	%	n	%	n	%	n	%
**Route of exposure**										
Inhalation/Nasal	2	(9.1)	3	(75.0)	8	(57.1)	140	(78.7)	153	(70.2)
Ingestion	19	(86.4)	1	(25.0)	6	(42.9)	28	(15.7)	54	(24.8)
Inhalation/Nasal/Ingestion	0	0.0	0	0.0	0	0.0	2	(1.1)	2	(0.9)
Rectal	0	0.0	0	0.0	0	0.0	1	(0.6)	1	(0.5)
Unknown	1	(4.5)	0	0.0	0	0.0	7	(3.9)	8	(3.7)
**Medical Outcome**										
Major effect	0	0.0	0	0.0	0	0.0	4	(2.2)	4	(1.8)
Moderate effect	4	(18.2)	1	(25.0)	5	(35.7)	90	(50.6)	100	(45.9)
Minor effect	4	(18.2)	1	(25.0)	4	(28.6)	45	(25.3)	54	(24.8)
No effect	1	(4.5)	1	(25.0)	1	(7.1)	8	(4.5)	11	(5.0)
Unable to follow, judged potentially toxic	11	(50.0)	1	(25.0)	3	(21.4)	24	(13.5)	39	(17.9)
Not followed, minimal or unrelated effects	2	(9.1)	0	0.0	1	(7.1)	7	(3.9)	10	(4.6)
**Intent**										
Intentional	0	0.0	2	(50.0)	12	(85.7)	162	(91.0)	176	(80.7)
Unintentional	21	(95.5)	2	(50.0)	2	(14.3)	6	(3.4)	31	(14.2)
Other	1	(4.5)	0	0.0	0	0.0	7	(3.9)	8	(3.7)
Unknown	0	0.0	0	0.0	0	0.0	3	(1.7)	3	(1.4)
**Caller location**										
Healthcare Facility	10	(45.5)	3	(75.0)	9	(64.3)	150	(84.3)	172	(78.9)
Own residence	9	(40.9)	1	(25.0)	5	(35.7)	22	(12.4)	37	(17.0)
School	1	(4.5)	0	0.0	0	0.0	2	(1.1)	3	(1.4)
Other	2	(9.1)	0	0.0	0	0.0	4	(2.2)	6	(2.8)
**Exposure site**										
Own residence	19	(86.4)	4	(100.0)	14	(100.0)	165	(92.7)	202	(92.7)
Other residence	2	(9.1)	0	0.0	0	0.0	2	(1.1)	4	(1.8)
School	1	(4.5)	0	0.0	0	0.0	6	(3.4)	7	(3.2)
Unknown	0	0.0	0	0.0	0	0.0	5	(2.8)	5	(2.3)

© 2019 Whitehill JM et al. *JAMA Network Open.*
